# Monomodality versus Combined Therapy in Optic Pathway Gliomas—20-Year Experience from a Singapore Children’s Hospital

**DOI:** 10.3389/fsurg.2022.827675

**Published:** 2022-05-02

**Authors:** Jia Xu Lim, Enrica E.K. Tan, Lee Ping Ng, Wan Tew Seow, Kenneth T.E. Chang, Ru Xin Wong, Wen Shen Looi, David C.Y. Low, Sharon Y.Y. Low

**Affiliations:** ^1^ Neurosurgical Service, KK Women’s and Children’s Hospital, Singapore, Singapore; ^2^ Paediatric Haematology/Oncology Service, KK Women’s and Children’s Hospital, Singapore, Singapore; ^3^ Department of Neurosurgery, National Neuroscience Institute, Singapore, Singapore; ^4^ SingHealth Duke-NUS Neuroscience Academic Clinical Program, Singapore, Singapore; ^5^ Department of Pathology and Laboratory Medicine, KK Women’s and Children’s Hospital, Singapore, Singapore; ^6^ Department of Radiation Oncology, National Cancer Centre, Singapore, Singapore

**Keywords:** optic pathway glioma, low grade glioma, outcomes, optic chiasmatic gliomas, pediatric glioma

## Abstract

**Introduction:**

The treatment of pediatric optic pathway gliomas (OPG) is challenging. At present, most centers provide individualized treatment to maximize progression free survival (PFS) and minimize morbidity. We aim to report our experience in the management of pediatric OPG, and investigate factors associated with an increased duration of remission after treatment.

**Methods:**

This is a single-institution study approved by the hospital ethics board. A retrospective review of consecutive OPGs managed from 2000 to 2020 was performed. Patients were divided into those managed with monomodality treatment (MT) and those who received combined therapy (CT). MT included various forms of surgery, chemotherapy and radiotherapy given alone, while CT involves a combination of surgery and adjuvant chemotherapy and/or radiotherapy.

**Results:**

Twenty-two patients were selected for this study. They had 40 treatment cycles; and a total follow up duration of 194.8 patient-years. Most of them were male (63.6%) and presented with visual deficits (72.7%). The mean age at initial presentation was 65 months and majority (86.4%) had their tumors arising directly from the optic chiasm, with 77.3% with hypothalamic extension. One patient had Neurofibromatosis type I (4.5%). The most common histological diagnosis was pilocytic astrocytoma (90.9%), followed by pilomyxoid astrocytoma (9.1%). The 5- and 10- year PFS were 46.2% and 36.4% respectively, while the 5- and 10-year OS were both 100%. When accounting for treatment type, there were 24 treatment cycles with MT (60.0%) and 16 CT (40.0%). After adjustment, treatments with MT were shown to have a shorter mean duration of remission (MT: 45 ± 49, CT: 84 ± 79 months; *p* = 0.007). Cox regression curve plotted after adjusting for patient’s age at treatment demonstrated a significantly longer PFS in the CT group (*p* = 0.037).

**Conclusions:**

Our results suggest a significant survival benefit of CT over MT for affected patients due to the prolonged the duration of disease remission, for both primary and subsequent treatments. Nonetheless, we acknowledge that our study reflects the outcomes of treatment strategies that have evolved over time. We emphasize the need for collective efforts from a dedicated multidisciplinary team and international collaborations for better disease understanding.

## Introduction

Optic pathway gliomas (OPG) are the most common intrinsic primary brain tumor affecting the optic pathway. However, as part of all central nervous system (CNS) tumors, they are rare. Overall, they comprise approximately 1 to 2% of all CNS tumors, and account for up to 5% of pediatric brain tumors (1–4). Also, OPGs tend to present during the first decade of life and are particularly prevalent in children with neurofibromatosis type 1 (NF1) (2, 5). Despite significant improvements in disease understanding, the optimal treatment strategy for this neoplasm remains controversial (2, 6). Over time, several modalities have been described. These include observation, different surgical techniques, chemotherapy regimens and various radiation therapy methods. Nonetheless, there is yet to be a single approach that is curative. Examples of key hurdles responsible for the lack of treatment standardization include OPG’s unpredictable clinical course, and previously limited knowledge of its biology due to its rarity as a tumor (4, 7, 8). Consequently, most centers manage OPGs with individualized treatment via a multidisciplinary consensus, with the priority to maximize progression free survival (PFS), whilst minimizing patient morbidity.

Histologically, the majority of OPG are pilocytic astrocytomas which are typically low-grade neoplasms with good long-term prognosis (9). Nonetheless, a small subset of them have propensity for aggressive behavior and have been identified to have a slightly higher Ki-67 index of 2 to 3% (9). Due to their slow-growing quiescent nature, affected children have longer survival times. Five-year overall survival rates have been reported to be as high as 80% (6, 10–13). As a disease entity, OPG represents a long-term challenge, with a chronic, protracted course (14). As a result of expectant-based approaches in managing these patients, they are more likely to undergo multiple lines of treatment that involve variations of surgery and, or non-surgical modalities. Although each treatment is advocated with the best intentions, the patient’s quality of life (QOL) will be inevitably affected. This is especially so for school-going children whose daily routines may disrupted by intermittent periods of hospital visits, and or inpatient stays. Building on this, we hypothesize that the aim to prolong the duration of disease remission between each treatment cycle should be included as one of the objectives of treatment. There has been no previous study comparing firstly, the length of disease remission periods in between treatments; and next, if mono- or multimodality approaches have any impact on the duration of disease remission. The primary aim of this study is to report our institutional experience in the management of pediatric OPG. Secondary aims include corroborating our clinical outcomes with current literature, and collective assessment of factors associated with an increased duration of remission after each treatment cycle.

## Materials and Methods

### Study Design and Patient Demographics

This is a single institution, retrospective study approved by the hospital ethics board (Singhealth CIRB reference 2014/ 2079). All patients below 19 years of age diagnosed with OPG that is confirmed on neuroimaging, and or histology; and subsequently underwent treatment at KK Women’s and Children’s Hospital, are included. Exclusion criteria encompass the following: patients with other histological types of suprasellar tumors, and patients with incomplete clinical information or lost to follow-up. In addition, patients diagnosed in our unit but subsequently managed elsewhere are excluded.

### Overview of OPG Treatment Workflow

At our institution, OPG patients are managed with a core multi-disciplinary team that comprises of the neurosurgeon, neuro-ophthalmologist, endocrinologist, and medical oncologist. If indicated, additional inputs from the medical geneticist, neuroradiologist, and or the radiation oncologist are included. Baseline investigations include the following: magnetic resonance imaging (MRI) of the brain and whole spine, a hormonal profile to assess the hypothalamic-pituitary axis, and formal visual assessment. Choice of treatment for each patient is discussed at regular multi-disciplinary meetings involving designated sub-specialists, and an individualized treatment plan is devised. Overall, the aims are to delay visual deterioration, optimize hormonal profile in a growing child, in order to maintain QOL and prolong progression free survival (PFS) as long as possible. Under such circumstances, treatment of either mono-modality treatment (MT) or combined treatments (CT) may be offered. For the purposes of this study, we define ‘MT’ as a single form of therapy given by itself, while ‘CT’ involves a combination of treatments given in continuity over a designated period. An example of such includes surgery of the OPG followed by adjuvant chemotherapy, and or radiotherapy. That is, the administration of MT or CT is only applicable to each individual treatment cycle. With regards to the type of surgery performed, (either biopsy or varying extents of resection), the neurosurgeon’s aims are to reduce raised intracranial pressure and local mass effect, in tandem with the preservation of neurological function. For example, OPGs involving only the optic nerve will be biopsied rather than resected (15); while those intimately close to the hypothalamic and optic apparatus will be debulked in keeping with the goals of maximal safe resection. Post-treatment followup is held conscientiously at regular intervals; initially at 3-monthly for the first year, and 6-monthly thereafter, depending on the patient’s condition and results of his/ her surveillance neuroimaging.

### Variables of Interest in this Study

Individual patients’ medical history, operative notes and radiological records are reviewed to identify variables such as age of diagnosis, gender, ethnicity, clinical presentation (visual deficit, other neurological deficits, endocrine dysfunction, raised intracranial pressure), treatment modality used, radiological features, histological diagnosis, treatment-related complications, and tumour recurrence rates. Here, we define ‘endocrine dysfunction’ as the presence of one or more hormonal abnormality based on investigations performed during the patient’s initial workup.The Dodge Classification is used to categorise each OPG’s proximity to the optic chiasm, and or hypothalamus in accordance with neuroimaging (16). In addition, we include the variable ‘Ki-67 index ≥2%’ to assess if affected patients required more lines of treatment. For the purposes of this study, we define ‘primary treatment’ as the initial treatment each patient underwent, while ‘subsequent treatment’ refers to the next treatment administered. Following that, patients and each of their treatment cycles are divided into those who underwent MT and those who undergo CT. Here, a treatment cycle is defined as the initiation of treatment modality (either MT or CT) and its subsequent followup period, until disease progression or tumor recurrence occurs. Next, we define this study’s outcome measures to be the following: ophthalmological function, endocrinological status, presence of obesity (body mass index of ≥30), and duration of remission after each treatment cycle. Each patient’s 5- and 10-year PFS and OS are also calculated. The PFS and OS are calculated from the initiation of primary treatment, whilst the duration of remission is calculated after every treatment cycle.

### Statistical Analysis

Statistical analyses are generated using SPSS version 20 (Statistical Package for the Social Sciences Statistics, Version 20.0; IBM, New York). Selected variables are described using frequency, with mean and standard deviation or median and interquartile range for categorical and continuous, respectively. Of note, the 2 main variables of interest, MT and CT are compared with either Pearson’s chi-square test or Fisher’s exact test where appropriate: and via 2 sample t-test or Mann-Whitney U test, depending on normality assumption. Categorical variables are reported as frequencies, and continuous variables are reported as mean with standard deviation (SD). Multivariable analysis and Cox regression curves for PFS and the duration of remission are conducted after adjustment for confounders have been identified from the univariate analysis. Unadjusted and adjusted odds ratio (ORs) with the 95% confidence intervals are reported. A *p*-value of <0.05 is considered statistically significant for this study.

## Results

**Figure 1:**
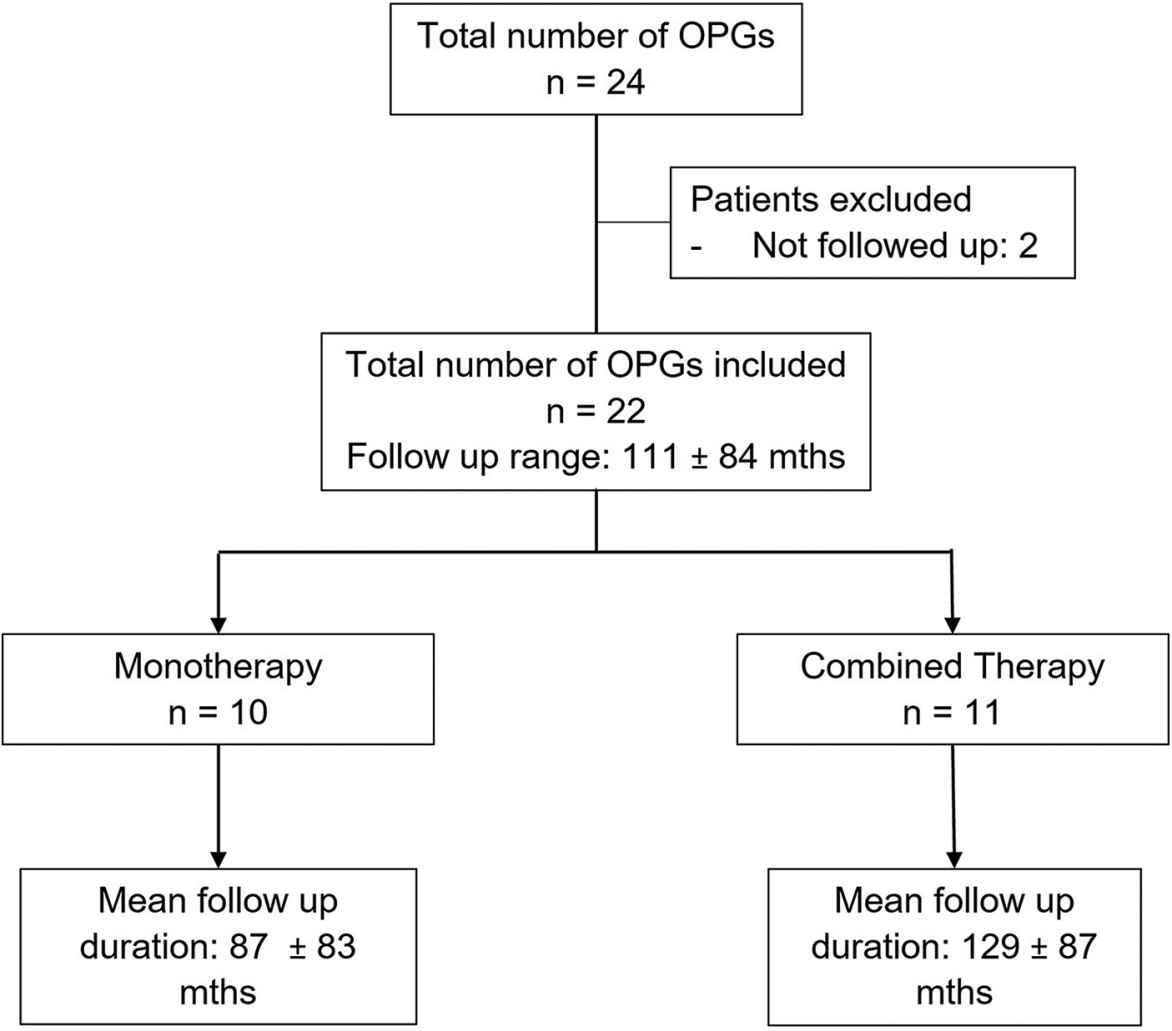
Overview of study population.

From 01 January 2000 to 31 December 2020, there were 24 patients diagnosed with OPG at our institution. However, 2 patients were excluded in the analysis. These included 1 patient who demised after surgery due to complications of hypernatremia, and another patient who was lost to followup. In the final study cohort of 22 patients, there were 10 patients (45.5%) who received MT and 12 patients (54.5%) who had CT. Both patient groups underwent a total of 40 treatment cycles: MT group had 24 (60.0%), whereas the CT group had 16 (40.0%) treatment cycles respectively. There was only 1 patient with NF1 in our series (4.5%). All patients were followed up for a mean duration of 9.3 years (111 months), for a total of 194.8 patient-years (2337 patient-months) (**Figure 1**).

### Overview of Patient Demographics, Tumor Features and Disease Management

The following variables of interest did not show statistical significance for both MT and CT groups: mean age at presentation, gender, ethnicity, presence of visual deficit, endocrine dysfunction, raised intracranial pressure, and neurological deficit at first diagnosis. We observed that both groups had a similar proportion of patients with OPGs centered at the chiasm (MT, 70.0%; CT, 100%) and with hypothalamic extension (MT, 70.0%; CT, 83.3%). Only one patient had metastatic disease (underwent CT) and one patient had NF-1 (underwent MT) in the series. This information is presented in **Table 1**. In terms of histological diagnosis, both MT and CT groups had a similar proportion of pilocytic astrocytoma (MT 90.0%; CT 91.7%) and pilomyxoid astrocytoma (MT 10.0%; CT 8.3%). Interestingly, the only 2 patients with Ki-67 index ≥2% were in the CT group (9.1%). In our series, only 6 patients (27.3%) with 8 treatment cycles (25.0%) underwent molecular sequencing, with 4 patients (66.7%) having 4 treatment cycles (50.0%) positive for BRAF mutation.

**Table 1:** Patient characteristics grouped by primary treatment.

	Overall (%)	Monotherapy (%)	Combined Therapy (%)	OR (95% CI)	*p*-value
Total number of OPGs	22 (100)	10 (45.5)	12 (54.5)	–	–
Follow up durations, months, mean ± SD	111 ± 84	87 ± 83	130 ± 83	–	0.26
Demographics					
Age, months, mean ± SD	65 ± 55	78 ± 56	55 ± 55	–	0.36
Male gender	14 (63.6)	7 (70.0)	7 (58.3)	–	0.68
Chinese race	10 (45.5)	5 (50.0)	5 (41.7)	–	0.70
Neurofibromatosis type 1	1 (4.5)	1 (10.0)	0 (0)	–	0.46
Clinical presentation					
Visual deficit	16 (72.7)	7 (70.0)	9 (75.0)	–	1.00
Endocrine dysfunction	1 (4.5)	0 (0)	1 (8.3)	–	1.00
Raised ICP	10 (45.5)	5 (50.0)	5 (41.7)	–	0.70
Neurological deficit	8 (36.4)	3 (30.0)	5 (41.7)	–	0.68
Tumour characteristics (Dodge Classification)					
Pre-chiasmatic	3 (13.6)	3 (30.0)	0 (0)	2.71 (1.51–4.89)	0.078
Chiasmatic	19 (86.4)	7 (70.0)	12 (100)	0.37 (0.20–0.66)	0.078
Post-chiasmatic	0 (0)	0 (0)	0 (0)	–	–
Hypothalamic extension	17 (77.3)	7 (70.0)	10 (83.3)	–	0.62
Presence of metastasis	1 (4.5)	0 (0)	1 (8.3)	–	1.00
Management					
Biopsy	2 (9.1)	1 (10.0)	1 (8.3)	–	1.00
Resection	19 (86.4)	8 (80.0)	11 (91.7)	–	0.57
Chemotherapy	8 (36.4)	0 (0)	8 (66.7)		**0.002**
Radiation therapy	7 (31.8)	2 (20.0)	5 (41.7)	–	0.38
CSF diversion procedure	14 (63.6)	4 (40.0)	10 (83.3)	0.13 (0.02–0.96)	0.074
More than 1 treatment cycle (after first treatment)	10 (45.5)	5 (50.0)	5 (41.7)	–	0.70
Histological diagnosis					
Pilocytic astrocytoma	20 (90.9)	9 (90.0)	11 (91.7)	–	1.00
Pilomyxoid astrocytoma	2 (9.1)	1 (10.0)	1 (8.3)	–	1.00
Ki-67 index ≥2%	2 (9.1)	0 (0)	2 (16.7)	–	0.48

*Bold values indicate statistically significant p-values <0.05.*

With regards to management, the MT group had a lower proportion of patients with chemotherapy as part of the first-line treatment (MT: 0%, CT: 66.7%; *p* =  0.011). In contrast, the proportions of patients that with other modalities (i.e., biopsy, resection, and radiation therapy) were similar. Also, we noted that a higher number of patients in the CT group required CSF diversion procedures (MT 40.0% versus CT 83.3%). However, there was no statistical significance between both groups after subsequent analysis (**Table 1**).

### Patient Outcomes

**Figure 2:**
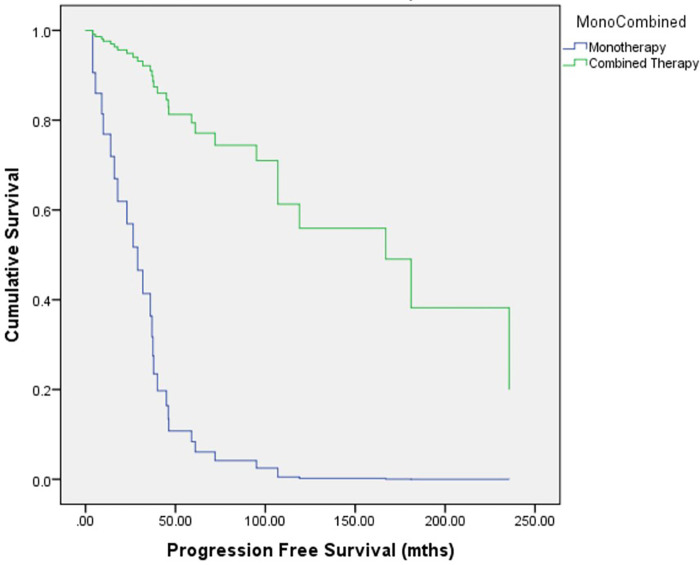
Cox regression curve for depicting duration of disease remission.

When considering outcomes based on the patient’s first-line treatment, both groups demonstrated similar rates of visual and endocrinological dysfunction, obesity, and mortality at their last followup. Although the adjusted mean progression free survival duration was longer in the CT group (MT 21 months versus CT 94 months, *p* = 0.002), the 5- and 10-year survival rate were similar with both groups at 100% at both timepoints (**Table 2**). With regards to outcomes based on treatment cycles, an association with NF-1, location of OPG, hypothalamic extension, presence of metastasis, and the histological diagnosis and the presence of Ki-67 ≥2% were also comparable. In our study, the MT group had a larger mean age at treatment (MT 112 months versus CT 62 months, *p* = 0.029) and were also less likely to have received surgical resection (MT: 60.0% versus CT: 93.3%; OR 0.11 [0.01–0.95], *p* = 0.030) and chemotherapy when compared to CT (MT: 20.0 versus CT: 66.7%; OR 0.13 [95% CI 0.03–0.54], *p* = 0.003). Most of our patients (86.7%) received chemotherapy according to the low-grade glioma protocol (COG A9952). There were 2 patients (13.3%) who received targeted therapy in the form of BRAF inhibitors; 1 patient was prescribed dabrafenib as MT, and another who had adjuvant trametinib after surgical debulking (hence, considered as CT). Of note, all but one treatment cycle within CT (*n* = 15) involves surgical resection, followed by adjuvant radiotherapy (*n* = 5), chemotherapy (COG A9952 protocol, *n* = 8; BRAF inhibitor, *n* = 1). The single case of biopsy was followed up with both adjuvant chemotherapy and radiotherapy. The duration in remission, after adjustment with age, surgical resection, the use of chemotherapy and the if it was primary or secondary treatment, was significantly longer in patients with CT (MT 45 months versus CT 87 months, *p* < 0.001). Using Cox regression curve, the PFS was also significantly less for MT (*p* = 0.003). These findings are presented in **Table 3** and **Figure 2**.

**Table 2:** Patient outcomes grouped by type of primary treatment.

	Overall (%)	Monotherapy (%)	Combined Therapy (%)	Unadjusted *p*-value	Adjusted *p*-value^a^
Visual worsening	11 (55.0)	3 (37.5)	8 (66.7)	0.36	0.36
Endocrinological worsening (from pre-treatment baseline)	15 (75.0)	5 (62.5)	10 (83.3)	0.35	0.30
Obesity	7 (38.9)	2 (33.3)	5 (41.7)	1.00	0.90
5-year PFS	6 (46.2)	1 (25.0)	5 (55.6)	0.56	–
10-year PFS	4 (36.4)	0 (0)	4 (57.1)	0.19	–
Progression free survival, months, mean ± SD	61 ± 74	21 ± 21	94 ± 85	**0.015**	**0.002**
5-year overall survival	13 (100)	4 (100)	9 (100)	–	–
10-year overall survival	11 (100)	4 (100)	7 (100)	–	–

^a^ *Adjusted according to tumour location and the use of chemotherapy.*

*Bold values indicate statistically significant p-values <0.05.*

**Table 3:** Treatment and duration of remission when grouped by type of treatment.

	Overall (%)	Monotherapy (%)	Combined Therapy (%)	OR (95% CI)	Unadjusted *p*-value	Adjusted *p*-value^a^
Total number of treatments	40 (100)	25 (62.5)	15 (37.5)	–	–	–
Follow up durations, months, mean ± SD	132 ± 81	132 ± 83	132 ± 81	–	0.99	–
Demographics						
Age at treatment, months, mean ± SD	93 ± 70	112 ± 73	62 ± 54		**0.029**	–
Male gender	24 (60.0)	15 (60.0)	9 (60.0)	–	1.00	–
Tumour characteristics (Dodge Classification)						
Neurofibromatosis type 1	2 (5.0)	1 (8.0)	0 (0)	–	0.52	–
Prechiasmatic	4 (10.3)	4 (16.7)	0 (0)	–	0.15	–
Chiasmatic	35 (89.7)	20 (83.3)	15 (100)			
Hypothalamic extension	30 (76.9)	17 (70.8)	13 (86.7)	–	0.44	–
Presence of metastasis	2 (5.1)	0 (0)	2 (13.3)	–	0.14	–
Management						
Biopsy	3 (7.5)	2 (8.3)	1 (6.3)	–	1.00	–
Resection	29 (72.5)	15 (60.0)	14 (93.3)	0.11 (0.01–0.95)	**0.030**	–
Chemotherapy	15 (37.5)	5 (20.0)	10 (66.7)	0.13 (0.03–0.54)	**0.003**	–
Radiation therapy	10 (25.0)	4 (16.0)	6 (40.0)	0.29 (0.07–1.26)	0.14	–
Primary treatment	22 (55.0)	10 (40.0)	12 (80.0)	0.17 (0.04–0.75)	**0.022**	–
Secondary treatment	18 (45.0)	15 (60.0)	3 (20.0)			
Histological diagnosis						
Pilocytic astrocytoma	33 (82.5)	21 (84.0)	12 (80.0)	–	1.00	–
Pilomyxoid astrocytoma	6 (15.0)	4 (16.0)	2 (13.3)	–	1.00	–
Ki-67 index ≥2%	4 (10.0)	1 (4.0)	3 (20.0)	–	0.14	–
Outcome						
Duration in remission, months, mean ± SD	61 ± 65	45 ± 49	87 ± 80	–	0.078	**<0.001**

^a^ *Adjusted according to age at time of treatment and the use of chemotherapy.*

*Bold values indicate statistically significant p-values <0.05.*

## Discussion

Pediatric OPG are extremely challenging tumours to manage. Over the years, various treatment modalities have been previously reported. However, there remains no global consensus on the best approach for affected children. Moreover, we are also now cognizant of the limitations of certain therapies. For example, the role of aggressive surgery for OPG is currently deemed less favorable, in view of the risks of iatrogenic injury to the hypothalamus, surrounding vascular structures and the optic apparatus (4). Next, historical experience with the complications of radiation treatment have led to preferential delay in its utilization, unless other options have be exhausted (17). In recent years, there is a paradigm shift towards the use of targeted therapy such as BRAF and MEK inhibitors (18, 19); and modern neurosurgical adjuncts for safer surgery via endoscopic approaches (20).

Good prognostic factors associated with OPG include younger age, NF-1 status, tumor location and extension, and histological diagnosis of pilomyxoid astrocytoma (6, 21–25). Conversely, intracranial hypertension and diencephalic syndrome at presentation, and tumour progression during followup are reported to be associated with poorer outcomes (26). In corroboration with other published studies, our patient cohort demonstrated some of these factors. For example, 31.8% of them presented at age less than 2 years old (mean age 65 months). There was 4.5% of patients with NF-1, 77.3% of the patients had OPG with hypothalamic extension; and 9.1% of the histological samples were pilomyxoid astrocytoma. Nonetheless, these factors did not demonstrate statistical significance for outcomes in our study.

### Progression Free Survival (PFS) and Overall Survival (OS)

In our series, 59.1% of all patients (*n* = 13) had more than 5 years of follow up, and 50% had 10 years of followup (*n* = 11). The 5- and 10-year PFS of our series was 46.2% and 36.4% respectively. This is comparatively less compared to the literature, where the PFS was reported to range from 44 to 71% for 5-years, and 46 to 58% for 10-years (6, 8, 10, 11, 13). We believe that one reason this may be the case is because of a higher number of patients in the MT group being treated with surgery alone as their primary treatment (80%). Broadly speaking, surgery alone as the primary treatment is controversial due to the inability for gross total resection without causing significant visual deficits. In our series, it has demonstrated, contrary to that in the literature, inferior PFS. When only CT is considered, the 5- and 10-year PFS of 56% and 57%, respectively, becomes commensurate with that reported.

Conversely, we report 100% 5- and 10-year OS in our series. This contrasts with the 5-year OS in the range of 80% and 10-year OS ranging from 46 to 78% reported in the literature (6, 10–13). This might be related to the multiple treatment cycles underwent by each patient and a long duration of remission between each cycle. Almost half of the patients in this series (45.5%) underwent more than 1 treatment cycle, with 2 patients with three treatments and 3 patients with four treatments. Of these secondary treatments, half were CT. Furthermore, the duration of remission between each treatment cycle was also significantly longer for CT treatment cycles (MT: 45 ± 49 months, CT: 84 ± 79 months; *p* = 0.007).

### Treatment Approaches and Outcomes

There are 2 positive findings in our study: firstly, a high OS at 5 and 10 years (100%); and that CT demonstrated a significantly longer remission when compared to MT, despite similar rates of visual and endocrinological dysfunction, and obesity amongst both groups. Following that, our series’ long-term data suggests that the duration of remission after each treatment cycle is equally important as the PFS after primary treatment. Of note, we are also able to lengthen disease remission without increased risks of complications, in a subset of our patients via surgical resection with adjuvant therapy.

As abovementioned, the majority of CT patients (93.3%) in our series, both as primary or subsequent therapies, involve surgical resection followed by either radiotherapy, and or chemotherapy. Our study demonstrates that such an approach appears to prolong the duration of disease remission between each treatment. This is correlative with similar rates of obesity, visual and endocrinological deficit at the last followup for our patients. Although aggressive resection carries risks of injury to the surrounding visual apparatus, hypothalamus and vascular structures, there are studies that report satisfactory neurosurgical outcomes (13, 27, 28). For example, Liu et al. reports that cytoreduction followed by adjuvant therapy is beneficial for local control. conferring longer OS and PFS compared with those who did not (13). Nonetheless, we believe that further prospective studies should be undertaken to investigate the feasibility of such an approach in a larger group.

### Role of Surgery in Optic Pathway Gliomas: Benefit or Pitfall?

The main emphasis in the last decade has been on chemotherapy as a first-line treatment for symptomatic or progressive tumors, particularly in younger children (29, 30). At present, the consensus is shifted towards less aggressive approaches because of the recognition of surgical risks, such as iatrogenic endocrine, visual, or hypothalamic dysfunction. In addition, OPGs are often diffusely infiltrative lesions, and the lack of clear margins makes complete resection challenging even when function can be preserved (31) Nevertheless, a proportion of OPGs will grow, even with maximal chemo-radiotherapeutic treatment. As such, it is suggested that some OPGs may benefit from surgery although the exact timing and its role is not universally accepted (31). There is a body of literature that demonstrate primary surgical debulking alone can be effective at controlling tumor growth and preserving function, without significant levels of postoperative morbidity (13, 14, 31). We acknowledge that 90% of the MT group in our study received surgery without adjuvant treatment. Upon review, these results are likely a combination of the following: firstly, some of them were operated in the very early years of our practice when disease understanding was poorer; multi-disciplinary discussions and therapeutic guidelines were not well-established; next, there was reluctance from some caregivers to commence chemotherapy owing to perceived side effects and opted for a ‘watch and wait’ approach. For them, agreement for adjuvant therapy was only administered after clinico-radiological tumor progression. Nonetheless, we are now cognizant of the updated evidence-based guidelines, including a more conservative approach to OPGs. Our current institutional practice is for all brain tumor cases to be discussed at multi-disciplinary meetings for consensus on the treatment approach for individual patients. Following that, a thorough discussion with patients and their caregivers on the natural history and recommendations will be arranged by our neurooncology team.

### Study Critique and Future Work

The authors acknowledge that there are limitations that should be highlighted. First and foremost, this is a study limited by its retrospective design and modest sample size. We agree that the study population is small; hence, on a larger scale, statistical conclusions may not be meaningful. However, our decision for a multivariable analysis aims to minimize, part of the confounders encountered. Furthermore, an analytical approach to our data can provide a better perspective and more objective assessments of any differences within our results. Subsequently, these findings aim to add to the growing body of literature and may be incorporated into future meta-analysis efforts, if needed. Separately, when analyzing patients based on treatment cycles (as depicted in **Table 2**), we are unable to definitively adjust for the type of previous treatment (i.e., surgical resection, chemotherapy, or radiotherapy) for treatment cycles with secondary treatment. This was mitigated by both the MT and CT groups having a similar proportion of treatment cycles with secondary treatment. Also, we are aware that the effect of targeted therapy, such as BRAF inhibitors is inadequately assessed due to the low usage in our series, especially in the early years before 2016. Nonetheless, our institution has since integrated molecular testing as part of the routine histopathology for pediatric brain tumors. Part of this investigation includes analysis for BRAF mutations. Next, there is a lack of available data on the neurocognitive, functional and QOL outcomes in our patient cohort. We believe this is particularly important in OPG patients with good OS, where they must live with the burden of any treatment complications from our clinical management. As part of our ongoing future work, we endeavor to followup on long-term patient outcomes beyond clinical assessments, such as delayed effects on neuropsychological development on these children.

## Conclusion

The management of pediatric OPG requires ongoing collaborative efforts to elucidate the optimal treatment strategy. Our study contributes to the current body of literature, especially from the perspective of an academic, tertiary institution based in Southeast Asia. In meantime, we strongly advocate international collaborations with like-minded individuals, to work towards better disease understanding for affected children.

## Data Availability

The raw data supporting the conclusions of this article will be made available by the authors upon reasonable request.

## References

[1] Shamji MF Benoit BG. Syndromic and sporadic pediatric optic pathway gliomas: review of clinical and histopathological differences and treatment implications. Neurosurg Focus. (2007) 23(5):E3. 10.3171/foc.2007.23.5.4 18004965

[2] Fried I Tabori U Tihan T Reginald A Bouffet E. Optic pathway gliomas: a review. CNS Oncol. (2013) 2(2):143–59. 10.2217/cns.12.47 25057976 PMC6169473

[3] Louis DN Ohgaki H Wiestler OD Cavenee WK Ellison DW Figarella-Branger D WHO classification of tumours of the central nervous system. Lyon: IARC (2016). 10.1007/s00401-016-1545-1 27157931

[4] Aihara Y Chiba K Eguchi S Amano K Kawamata T. Pediatric optic pathway/hypothalamic glioma. Neurol Med Chir (Tokyo). (2018) 58(1):1–9. 10.2176/nmc.ra.2017-0081 29118304 PMC5785691

[5] Shofty B Ben Sira L Constantini S. Neurofibromatosis 1-associated optic pathway gliomas. Childs Nerv Syst. (2020) 36(10):2351–61. 10.1007/s00381-020-04697-1 32524182

[6] Hidalgo ET Kvint S Orillac C North E Dastagirzada Y Chang JC Long-term clinical and visual outcomes after surgical resection of pediatric pilocytic/pilomyxoid optic pathway gliomas. J Neurosurg Pediatr. (2019) 24(2):166–73. 10.3171/2019.2.PEDS18529 31100719

[7] Parsa CF Hoyt CS Lesser RL Weinstein JM Strother CM Muci-Mendoza R Spontaneous regression of optic gliomas: thirteen cases documented by serial neuroimaging. Arch Ophthalmol. (2001) 119(4):516–29. 10.1001/archopht.119.4.516 11296017

[8] Nicolin G Parkin P Mabbott D Hargrave D Bartels U Tabori U Natural history and outcome of optic pathway gliomas in children. Pediatr Blood Cancer. (2009) 53(7):1231–7. 10.1002/pbc.22198 19621457

[9] Cummings TJ Provenzale JM Hunter SB Friedman AH Klintworth GK Bigner SH Gliomas of the optic nerve: histological, immunohistochemical (MIB-1 and p53), and MRI analysis. Acta Neuropathol. (2000) 99(5):563–70. 10.1007/s004010051161 10805102

[10] Khafaga Y Hassounah M Kandil A Kanaan I Allam A El Husseiny G Optic gliomas: a retrospective analysis of 50 cases. Int J Radiat Oncol Biol Phys. (2003) 56(3):807–12. 10.1016/s0360-3016(02)04512-1 12788189

[11] Ater JL Zhou T Holmes E Mazewski CM Booth TN Freyer DR Randomized study of two chemotherapy regimens for treatment of low-grade glioma in young children: a report from the Children’s Oncology Group. J Clin Oncol. (2012) 30(21):2641–7. 10.1200/JCO.2011.36.6054 22665535 PMC3413276

[12] El Beltagy MA Reda M Enayet A Zaghloul MS Awad M Zekri W Treatment and Outcome in 65 Children with Optic Pathway Gliomas. World Neurosurg. (2016) 89:525–34. 10.1016/j.wneu.2016.02.042 26898488

[13] Liu Y Hao X Liu W Li C Gong J Ma Z Analysis of survival prognosis for children with symptomatic optic pathway gliomas who received surgery. World Neurosurg. (2018) 109:e1–e15. 10.1016/j.wneu.2017.09.144 28986229

[14] Goodden J Pizer B Pettorini B Williams D Blair J Didi M The role of surgery in optic pathway/hypothalamic gliomas in children. J Neurosurg Pediatr. (2014) 13(1):1–12. 10.3171/2013.8.PEDS12546 24138145

[15] Qiu L Chigurupathi S Seow WT Yy Low S. Intraoperative visual evoked potential monitoring for optic pathway glioma in an infant: A case description. Clin Neurophysiol. (2020) 131(8):1772–4. 10.1016/j.clinph.2020.05.007 32504939

[16] Dodge HW Jr. Love JG Craig WM Dockerty MB Kearns TP Holman CB Gliomas of the optic nerves. AMA Arch Neurol Psychiatry. (1958) 79(6):607–21. 10.1001/archneurpsyc.1958.02340060003001 13532071

[17] Merchant TE Conklin HM Wu S Lustig RH Xiong X. Late effects of conformal radiation therapy for pediatric patients with low-grade glioma: prospective evaluation of cognitive, endocrine, and hearing deficits. J Clin Oncol. (2009) 27(22):3691–7. 10.1200/JCO.2008.21.2738 19581535 PMC2799064

[18] Thomas RP Gibbs IC Xu LW Recht L. Treatment options for optic pathway gliomas. Curr Treat Options Neurol. (2015) 17(2):333. 10.1007/s11940-014-0333-2 25619537

[19] Banerjee A Jakacki RI Onar-Thomas A Wu S Nicolaides T Young Poussaint T A phase I trial of the MEK inhibitor selumetinib (AZD6244) in pediatric patients with recurrent or refractory low-grade glioma: a Pediatric Brain Tumor Consortium (PBTC) study. Neuro Oncol. (2017) 19(8):1135–44. 10.1093/neuonc/now282 28339824 PMC5570236

[20] Bin Abdulqader S Al-Ajlan Z Albakr A Issawi W Al-Bar M Recinos PF Endoscopic transnasal resection of optic pathway pilocytic astrocytoma. Childs Nerv Syst. (2019) 35(1):73–81. 10.1007/s00381-018-3994-4 30338361

[21] Chan MY Foong AP Heisey DM Harkness W Hayward R Michalski A. Potential prognostic factors of relapse-free survival in childhood optic pathway glioma: a multivariate analysis. Pediatr Neurosurg. (1998) 29(1):23–8. 10.1159/000028680 9755308

[22] Opocher E Kremer LC Da Dalt L van de Wetering MD Viscardi E Caron HN Prognostic factors for progression of childhood optic pathway glioma: a systematic review. Eur J Cancer. (2006) 42(12):1807–16. 10.1016/j.ejca.2006.02.022 16809032

[23] Jeon YK Cheon JE Kim SK Wang KC Cho BK Park SH. Clinicopathological features and global genomic copy number alterations of pilomyxoid astrocytoma in the hypothalamus/optic pathway: comparative analysis with pilocytic astrocytoma using array-based comparative genomic hybridization. Mod Pathol. (2008) 21(11):1345–56. 10.1038/modpathol.2008.88 18622384

[24] Rodriguez FJ Lim KS Bowers D Eberhart CG. Pathological and molecular advances in pediatric low-grade astrocytoma. Annu Rev Pathol. (2013) 8:361–79. 10.1146/annurev-pathol-020712-164009 23121055 PMC3600584

[25] Dodgshun AJ Elder JE Hansford JR Sullivan MJ. Long-term visual outcome after chemotherapy for optic pathway glioma in children: Site and age are strongly predictive. Cancer. (2015) 121(23):4190–6. 10.1002/cncr.29649 26280460

[26] Rakotonjanahary J De Carli E Delion M Kalifa C Grill J Doz F Mortality in children with optic pathway glioma treated with up-front BB-SFOP chemotherapy. PLoS One. (2015) 10(6):e0127676. 10.1371/journal.pone.0127676 26098902 PMC4476571

[27] Gillett GR Symon L. Hypothalamic glioma. Surg Neurol. (1987) 28(4):291–300. 10.1016/0090-3019(87)90309-0 3629459

[28] Wisoff JH Abbott R Epstein F. Surgical management of exophytic chiasmatic-hypothalamic tumors of childhood. J Neurosurg. (1990) 73(5):661–7. 10.3171/jns.1990.73.5.0661 2213155

[29] Jahraus CD Tarbell NJ. Optic pathway gliomas. Pediatr Blood Cancer. (2006) 46(5):586–96. 10.1002/pbc.20655 16411210

[30] Massimi L Tufo T Di Rocco C. Management of optic-hypothalamic gliomas in children: still a challenging problem. Expert Rev Anticancer Ther. (2007) 7(11):1591–610. 10.1586/14737140.7.11.1591 18020927

[31] Hill CS Khan M Phipps K Green K Hargrave D Aquilina K. Neurosurgical experience of managing optic pathway gliomas. Childs Nerv Syst. (2021) 37(6):1917–29. 10.1007/s00381-021-05060-8 33532921 PMC8184710

